# Clinical Outcomes of Rubber‐clip‐assisted Versus Conventional Colorectal Endoscopic Submucosal Dissection: A Retrospective Study

**DOI:** 10.1002/deo2.70415

**Published:** 2026-09-23

**Authors:** Kohei Uyama, Hiroyoshi Iwagami, Takuji Akamatsu, Riki Sakano, Tomoya Kitada, Masayuki Shimoyama, Tomoko Terashita, Yasuki Nakatani, Yoshito Uenoyama, Yukitaka Yamashita

**Affiliations:** ^1^ Department of Gastroenterology and Hepatology Japanese Red Cross Wakayama Medical Center Wakayama Japan; ^2^ Akamatsu Gastroenterology, Hepatology and Endoscopy Clinic Wakayama Japan

**Keywords:** colorectal cancer, endoscopic submucosal dissection, perforation, procedure time, traction

## Abstract

**Objectives:**

Adequate traction is essential for safe and efficient colorectal endoscopic submucosal dissection (ESD), but gravitational traction is often insufficient in certain situations. Several traction techniques are reportedly effective. We use the rubber‐clip method (RCM), which is a simple and inexpensive technique that combines a standard clip with a rubber band to allow directional traction adjustment. This study was performed to evaluate the clinical outcomes of RCM‐assisted colorectal ESD.

**Methods:**

Among 551 colorectal ESD procedures performed between January 2014 and October 2025, 452 patients were eligible for inclusion. Propensity score matching using seven covariates yielded 56 RCM and 56 non‐RCM cases. The primary endpoint was procedure time, and the secondary endpoints were perforation, bleeding, en bloc resection, and pathological complete resection. Additionally, all procedures were classified as long‐duration (≥120 min) or short‐duration (<120 min) to identify independent risk factors for prolonged procedures, including intraoperative variables.

**Results:**

After matching, the RCM group showed a significantly shorter procedure time than the non‐RCM group (66.8 vs. 90.7 min, respectively; *p* = 0.024) and a lower perforation rate (0.0 % vs. 10.7%, respectively; *p* = 0.027). In the multivariate analysis, absence of traction, non‐expert endoscopist, moderate‐to‐severe fibrosis, large lesion size, and poor maneuverability were independent risk factors for prolonged procedures, with absence of traction showing the strongest association.

**Conclusions:**

The RCM shortened procedure time and reduced perforation risk. Given its low cost, simplicity, and ability to adjust traction direction, the RCM may represent an effective traction method for colorectal ESD.

**Trial Registration**: N/A.

## 1 Introduction

Colorectal cancer ranks third in global incidence and second in cancer‐related mortality [1]. In many countries, the incidence of colorectal cancer has been increasing with lifestyle westernization, and the incidence of early‐onset colorectal cancer has been increasing by approximately 1%–4% annually [1]. Endoscopic screening and removal of adenomatous lesions are considered essential for reducing the incidence of colorectal cancer [1]. Endoscopic resection plays an essential role in reducing colorectal cancer‐related mortality because this approach lowers the risk to a level comparable to that of the general population [2].

Endoscopic mucosal resection (EMR) is widely used and generally safe; however, the cumulative incidence of gastrointestinal polyp recurrence at 3 months was 6.5% in the underwater EMR group and 7.0% in the conventional EMR group [3, 4]. Compared with EMR, endoscopic submucosal dissection (ESD) for colorectal tumors achieves higher en bloc resection and curative resection rates [5]. A large colorectal ESD cohort study reported 5‐year overall and disease‐specific survival rates of 94.6% and 100%, respectively, with a low local recurrence rate of 1.5% [6]. Despite these favorable outcomes, colorectal ESD remains technically demanding because of the thin intestinal wall, poor endoscope maneuverability, and relatively high risk of complications, particularly when the procedure time is prolonged [7, 8]. Although ESD is desirable for achieving en bloc resection in large or bulky lesions, these cases are especially prone to prolonged procedure time. To address this issue and perform safe, efficient dissection, traction plays a crucial role in shortening the procedure time by improving visualization and maintaining an appropriate submucosal plane.

Several traction methods, including the clip‐with‐line method, clip‐and‐snare method, Sakamoto‐Osada (S‐O) clip method, and multiloop technique, have been proposed [9, 10, 11, 12]. However, these traction techniques have several limitations, including the need for endoscope reinsertion or the requirement for dedicated devices, which may increase costs and restrict their availability to certain institutions. At our institution, we use the rubber‐clip method (RCM), in which a rubber band is attached to a standard clip. This technique provides traction without the need for endoscope reinsertion and is more cost‐efficient than previously described methods [13]. Moreover, the RCM allows adjustment of the traction direction during the procedure by cutting the rubber band and repositioning it when necessary. In the present study, we compared clinical outcomes and complications between RCM‐assisted colorectal ESD and conventional ESD.

## 2 Patients and Methods

### 2.1 Patients

This retrospective study included 551 consecutive colorectal ESD procedures performed at our institution between January 2014 and October 2025. The exclusion criteria were neuroendocrine tumors, procedures discontinued because of severe fibrosis or technical difficulty, and use of a traction method other than the RCM. The RCM was introduced at our institution in mid‐2016. Before its introduction, colorectal ESD was routinely performed without any traction device. Thereafter, the primary endoscopist adopted the RCM as the standard traction method for colorectal ESD. For lesions located in the lower rectum (Rb), a clip‐with‐line method was occasionally used; for all other colorectal locations, the RCM was consistently applied. The present study was performed in accordance with the principles of the Declaration of Helsinki. Institutional review board approval was obtained (1608). The rubber band used in this study is a commercially available polyurethane thread not originally manufactured as a medical device. Its off‐label use was approved by the institutional review board. However, the rubber band is removed together with the specimen at the end of the procedure to ensure that no foreign material remains in the body, and the patient's latex allergy status is always confirmed before the procedure. Given the retrospective design, the requirement for written informed consent was waived, and an opt‐out method was employed.

### 2.2 Evaluated Items

The included cases were categorized into RCM and non‐RCM groups. To compare treatment outcomes, we calculated a propensity score using age, sex, macroscopic type, lesion location, lesion size, endoscopist experience, and degree of fibrosis as covariates, followed by 1:1 matching. The primary endpoint was procedure time, and the secondary endpoints were the perforation rate, bleeding rate, en bloc resection rate, and pathological complete resection rate.

Univariate analysis was also performed to assess the association between prolonged procedure time (≥120 min) [14] and background factors, namely age, sex, macroscopic type, lesion location, lesion size, and the following treatment factors: traction, endoscope maneuverability, degree of fibrosis, and endoscopist experience. Variables significantly associated with prolonged procedure time in the univariate analysis, along with endoscope maneuverability, were entered into a multivariate logistic regression model to identify independent risk factors, including intraoperative variables.

### 2.3 Definitions of Outcomes, Pathological Evaluation, and Adverse Events

Procedure time was defined as the duration from the initial mucosal incision to the completion of lesion removal. En bloc resection was defined as removal of the lesion in a single piece without fragmentation, allowing accurate histopathological assessment of the resection margins. All resected specimens were fixed in formalin and serially sectioned at 2‐mm intervals for histopathological evaluation. Pathological complete resection was defined as en bloc resection with tumor‐free lateral and vertical margins. An expert was defined as an endoscopist certified as a board‐certified trainer by the Japan Gastroenterological Endoscopy Society with at least 10 years of clinical experience. Intraprocedural bleeding was defined as spurting blood or persistent oozing that did not stop spontaneously within 60 s or following water irrigation and required endoscopic hemostasis with endoclips or coagulation. Delayed bleeding was defined as postprocedural bleeding presenting with hematochezia or melena requiring endoscopic hemostasis and blood transfusion [15]. Intraprocedural perforation was defined as a visible defect in the muscularis propria detected endoscopically during the procedure. Delayed perforation was defined as colorectal perforation recognized with X‐rays or computed tomography after completion of ESD without intraprocedural or immediate postprocedural evidence of perforation [15]. Maneuverability was defined as the ease of controlling the endoscope to obtain a stable view and to approach the lesion with appropriate angles and distances throughout the procedure. Submucosal fibrosis was assessed endoscopically during ESD and classified into four grades: None, Mild, Moderate, and Severe. “None” indicates the absence of fibrosis, characterized by a transparent submucosal layer. “Mild” indicates fibrosis appearing as a white, web‐like structure within the submucosal layer. “Moderate” indicates more advanced fibrosis, characterized by a whitish muscular structure with loss of submucosal transparency. “Severe” refers to marked fibrosis presenting as a dense, scallop‐like appearance and is defined as fibrosis that led to either discontinuation of submucosal dissection or the occurrence of perforation. According to the grading system reported by Kim et al. [16], None corresponds to F0, Mild corresponds to F1, and Moderate and Severe correspond to F2.

### 2.4 ESD Procedure

**FIGURE 1:**
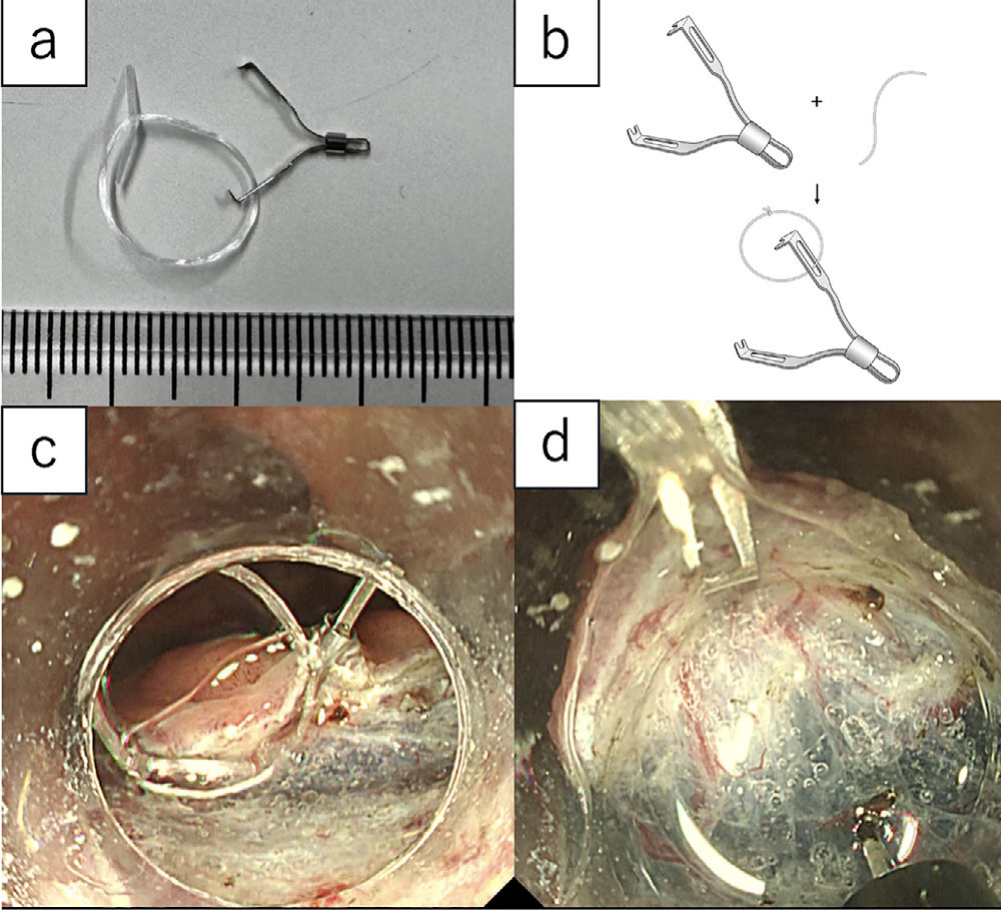
Rubber‐clip method in endoscopic submucosal dissection. (a, b) Clip with an attached rubber band. The rubber band is looped around the hemoclip arms before deployment. (c) The clip with the rubber band is attached to the lesion margin. (d) Traction is applied by anchoring the rubber band to the contralateral wall with a second clip, providing clear visualization of the submucosal plane during dissection.

ESD was performed using PCF‐H290ZI, PCF‐H290TI, or CF‐XZ1200I endoscopes (Olympus, Tokyo, Japan), with a DH‐30CR distal attachment (Olympus). A HookKnife (Olympus) was primarily used for mucosal incision and submucosal dissection. Procedures were performed by an expert and a non‐expert endoscopist together, with one endoscopist as the primary operator and the other assisting. Our standard strategy was to perform the RCM after completing circumferential incision and trimming. In this method, a clip with a rubber band (Figure 1) [13] is attached to the lesion margin, and the rubber band is anchored to the contralateral mucosa with another clip to provide effective traction.

Traction was applied using a polyurethane rubber band (Operon rubber thread, H639; diameter, 0.3 mm; 100% spandex; approximate cost, 300 JPY per 20‐m roll [MIYUKI Co., Ltd., Tokyo, Japan]). The band was looped around a standard hemoclip before deployment and anchored to the contralateral colonic wall with a second clip, providing continuous traction across the submucosal dissection plane (Figure 1).

### 2.5 Statistical Analysis

Statistical analyses were performed using EZR (Saitama Medical Center, Jichi Medical University, Saitama, Japan) [17]. Categorical data were compared using Fisher's exact probability test or the chi‐square test. Continuous data were compared using the Mann–Whitney U test. Associations between the evaluated factors and long procedure time were assessed using univariate and multivariate logistic regression. Variables significant in the univariate analysis and those that appeared important in daily practice were used in the multivariate analysis. *P* < 0.050 was considered statistically significant.

## 3 Results

### 3.1 Patient and Lesion Characteristics Before Propensity Score Matching

**FIGURE 2:**
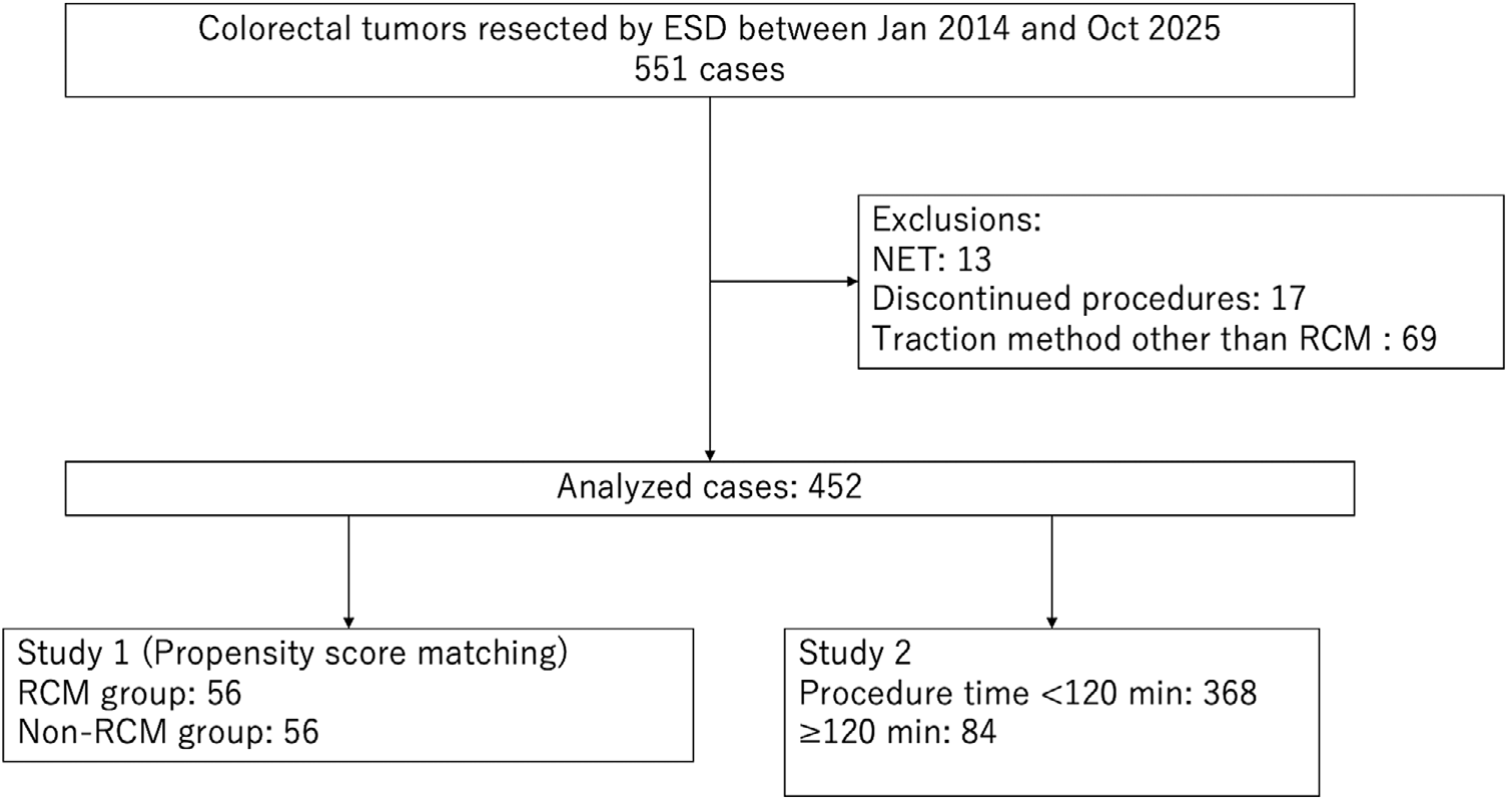
Flowchart of the present study. ESD, endoscopic submucosal dissection; NET, neuroendocrine tumor.

After excluding 13 neuroendocrine tumor cases, 17 discontinued procedures, and 69 cases in which other traction devices were used, 452 lesions were included in the final analysis (Figure 2). Among the 452 lesions, 56 were treated without traction (non‐RCM group) and 396 with the RCM. There were no significant differences between the RCM and non‐RCM groups in age, sex, macroscopic type, or lesion size. However, endoscopist experience differed significantly, with non‐expert endoscopists represented more frequently in the RCM group than in the non‐RCM group (*p* = 0.015). Lesion location also differed significantly between the groups (*p* = 0.008): rectal lesions were most common in the non‐RCM group (8/56), whereas ascending colon lesions were most common in the RCM group (101/396) (Table 1).

**TABLE 1:** Baseline characteristics of the patients in the rubber‐clip method (RCM) and non‐RCM groups before propensity score matching.

Parameter	Non‐RCM (*n* = 56)	RCM (*n* = 396)	*p*‐Value
Age, years	69.27 ± 10.02	70.05 ± 10.12	0.590
Sex			0.770
Female	26 (46.4)	173 (43.7)	
Male	30 (53.6)	223 (56.3)	
Lesion location			0.008
Cecum	4 (7.1)	59 (14.9)	
Ascending colon	8 (14.3)	101 (25.5)	
Transverse colon	8 (14.3)	80 (20.2)	
Descending colon	5 (8.9)	30 (7.9)	
Sigmoid colon	9 (16.1)	52 (13.1)	
Rectum	22 (39.3)	74 (18.7)	
Macroscopic classification			0.320
I	24 (42.9)	181 (45.7)	
IIa	32 (57.1)	200 (50.5)	
IIc	0 (0.0)	15 (3.8)	
Lesion size, mm	27.77 ± 9.64	31.52 ± 14.68	0.064
Endoscopist experience			0.015
Non‐expert	17 (30.4)	190 (48.0)	
Expert	39 (69.6)	206 (52.0)	
Fibrosis			0.051
None to mild	50 (89.3)	304 (76.8)	
Moderate to severe	6 (10.7)	92 (23.2)	

Data are presented as mean ± standard deviation or *n* (%).

RCM, rubber‐clip method.

Macroscopic classification was assessed according to the Paris classification of superficial neoplastic lesions.

### 3.2 Patient and Lesion Characteristics After Propensity Score Matching

After matching, 56 cases were included in each group (RCM vs. non‐RCM) (Figure 2 and Table 2). Following matching, the mean procedure time was significantly shorter in the RCM group (66.8 ± 52.4 min) than in the non‐RCM group (90.7 ± 57.4 min) (*p* = 0.024). The intraprocedural perforation rate was 10.7% (6/56) in the non‐RCM group and 0.0 % (0/56) in the RCM group, with a significant difference (*p* = 0.027). No significant differences were found in intraprocedural bleeding (0.0% vs. 1.8%; *p* = 1.000) or en bloc resection rates (98.2% vs. 92.9%; *p* = 0.364) (Table 2) between the RCM and non‐RCM groups, respectively (Table 2). The pathological complete resection rate was 85.7% (48/56) in the non‐RCM group and 94.6% (53/56) in the RCM group (*p* = 0.203). Most non‐RCM cases were concentrated in the earlier study period before the introduction of RCM, which may partly explain the higher perforation rate.

**TABLE 2:** Baseline characteristics and clinical outcomes in the rubber‐clip method (RCM) and non‐RCM groups after propensity score matching.

Parameter	Non‐RCM (*n* = 56)	RCM (*n* = 56)	*p*‐Value
Age, years	69.27 ± 10.02	69.80 ± 9.88	0.776
Sex			0.337
Female	26 (46.4)	20 (35.7)	
Male	30 (53.6)	36 (64.3)	
Macroscopic classification			0.705
I	24 (42.9)	27 (49.2)	
IIa	32 (57.1)	29 (51.8)	
Lesion location			0.559
Cecum	4 (7.1)	3 (5.4)	
Ascending colon	8 (14.3)	15 (26.8)	
Transverse colon	8 (14.3)	10 (17.9)	
Descending colon	5 (8.9)	4 (7.1)	
Sigmoid colon	9 (16.1)	9 (16.1)	
Rectum	22 (39.3)	15 (26.8)	
Lesion size, mm	27.77 ± 9.64	27.73 ± 11.29	0.986
Endoscopist experience			0.069
Non‐expert	17 (30.4)	8 (14.3)	
Expert	39 (69.6)	48 (85.7)	
Fibrosis			0.775
None to mild	50 (89.3)	48 (85.7)	
Moderate to severe	6 (10.7)	8 (14.3)	
**Outcome**			
Procedure time, min	90.7 ± 57.4	66.8 ± 52.4	0.024
Intraprocedural perforation	6 (10.7)	0 (0.0)	0.027
Intraprocedural bleeding	1 (1.8)	0 (0.0)	1.000
En bloc resection	52 (92.9)	55 (98.2)	0.364
Pathological complete resection	48 (85.7)	53 (94.6)	0.203

Data are presented as mean ± standard deviation or *n* (%).

RCM, rubber‐clip method.

Macroscopic classification was assessed according to the Paris classification of superficial neoplastic lesions.

### 3.3 Clinical Factors Associated With Prolonged Procedure Time

For the univariate and multivariate analyses, lesions were stratified by procedure time into the short (S) procedure time group (<120 min; *n* = 368) and long (L) procedure time group (≥120 min; *n* = 84) (Figure 2). Univariate analysis showed that absence of traction (*p* = 0.009), non‐expert endoscopist (*p* = 0.002), moderate‐to‐severe fibrosis (*p* < 0.001), and large lesion size (*p* < 0.001) were significantly more common in the L than S group. Endoscope maneuverability did not differ significantly between the groups (*p* = 0.140) (Table 3). Multivariate logistic regression analysis demonstrated that absence of traction, non‐expert endoscopist, moderate‐to‐severe fibrosis, large lesion size, and poor maneuverability were independent factors associated with prolonged procedure time (all *p* < 0.01). Among these factors, absence of traction had the strongest association with prolonged procedure time, with an odds ratio of 6.58 (95% confidence interval: 3.06–14.10) (Table 4).

**TABLE 3:** Univariate analysis comparing the S and L groups.

Variable	S (*n* = 368)	L (*n* = 84)	*p*‐Value
Age, years	63.75 (37–93)	65.75 (52–94)	0.099
Sex			1.000
Female	162 (44.0)	37 (44.0)	
Male	206 (56.0)	47 (56.0)	
Lesion location			0.440
Cecum	56 (15.2)	7 (8.3)	
Ascending colon	89 (24.2)	20 (23.8)	
Transverse colon	72 (19.6)	16 (19.0)	
Descending colon	27 (7.3)	8 (9.5)	
Sigmoid colon	51 (13.9)	10 (11.9)	
Rectum	73 (19.8)	23 (27.4)	
Macroscopic classification			0.980
I	167 (45.4)	38 (45.2)	
IIa	188 (51.1)	44 (52.4)	
IIc	13 (3.5)	2 (2.4)	
Lesion size, mm	20 (7–70)	30 (12–95)	<0.001
Traction			0.009
No	38 (10.3)	18 (21.4)	
Yes	330 (89.7)	66 (78.6)	
Maneuverability			0.140
Good	296 (80.4)	61 (72.6)	
Poor	72 (19.6)	23 (27.4)	
Fibrosis			<0.001
None to mild	326 (88.6)	60 (71.4)	
Moderate to severe	42 (11.4)	24 (28.6)	
Endoscopist experience			0.002
Non‐expert	155 (42.1)	52 (61.9)	
Expert	213 (57.9)	32 (38.1)	

Data are presented as median (range) or *n* (%).

L, long procedure time; S, short procedure time.

Macroscopic classification was assessed according to the Paris classification of superficial neoplastic lesions.

**TABLE 4:** Multivariate analysis of risk factors associated with prolonged procedure time (≥120 min) during endoscopic submucosal dissection.

Factor	Odds ratio (95% CI)	*p*‐Value
Non‐RCM	6.58 (3.06–14.10)	<0.001
Lesion size	1.09 (1.07–1.11)	<0.001
Moderate‐to‐severe fibrosis	5.22 (2.55–10.70)	<0.001
Poor maneuverability	2.13 (1.12–4.04)	<0.001
Non‐expert endoscopist	4.31 (2.31–8.00)	<0.001

CI, confidence interval; RCM, rubber‐clip method.

## 4 Discussion

Although rubber band‐assisted traction methods, including the orthodontic rubber band technique  [18] and the dual‐clip and rubber band method [19], shorten procedure time in colorectal ESD, previous studies involved small sample sizes. In contrast, the present study included a large number of cases, providing robust evidence supporting the clinical usefulness of traction. Moreover, the use of propensity score matching minimized background bias, which allowed comparisons between the RCM and non‐RCM groups.

Technical difficulty in colorectal ESD is influenced by various factors. Low‐volume centers and poor lifting of the lesion are linked to unsuccessful en bloc resection, while prolonged procedure time is associated with large lesion size, institutional volume, and endoscopist experience  [20]. Flexural lesions, scarred or recurrent tumors, and lesion size of ≥50 mm are predictors of prolonged procedure time, piecemeal resection, and increased risk of perforation [21]. Poor maneuverability and severe fibrosis also increase the risk of incomplete resection and perforation [22].

Traction‐assisted ESD has attracted attention as a means of enhancing visualization. Although its benefits are well established in esophageal and gastric ESD [23, 24], studies in the colon have shown inconsistent effects on procedure time  [25, 26]. However, some traction methods require reinsertion of the endoscope or rely on specialized devices, creating limitations related to cost and availability [27]. Reported outcomes vary among traction techniques. In one study, the multiloop method did not reduce dissection time, although it significantly increased dissection speed [27]. The S‐O clip shortened procedure time [11], and the clip‐and‐thread technique not only shortened procedure time, but also improved independent completion rates among intermediate‐level endoscopists [28].

Traction direction is an important determinant of effectiveness, and vertical traction is considered ideal [29, 30]. A randomized controlled trial in early gastric cancer demonstrated that vertical traction using the S‐O clip significantly reduced procedure time during ESD [31]. The RCM provides vertical traction regardless of lesion location, and the traction direction can be readjusted easily by cutting the rubber band and repositioning a new rubber band. Although similar adjustments are possible with the S‐O clip, the RCM may offer practical advantages in terms of simplicity and lower material cost because it does not require dedicated traction devices.

Recent studies further support the efficacy of rubber band‐assisted traction in colorectal ESD. After 1:1 propensity score matching, dual‐clip and rubber band‐assisted ESD achieved a significantly shorter procedure time compared with conventional ESD (30.0 vs. 44.0 min, respectively; *p* < 0.050) in 35 matched pairs [19]. Similarly, orthodontic rubber band‐assisted ESD demonstrated a significantly shorter procedure time compared with conventional ESD (35 vs. 50 min, respectively; *p* = 0.001) in 31 matched pairs [18]. Use of the RCM significantly shortened the procedure time and reduced perforation rates compared with non‐RCM procedures. These benefits may be attributed to improved visualization and clearer identification of the submucosal plane afforded by stable vertical traction. Furthermore, multivariate analysis identified absence of traction, non‐expert endoscopist, moderate‐to‐severe fibrosis, large lesion size, and poor maneuverability as independent risk factors for prolonged procedure time, with absence of traction exhibiting the strongest association.

**FIGURE 3:**
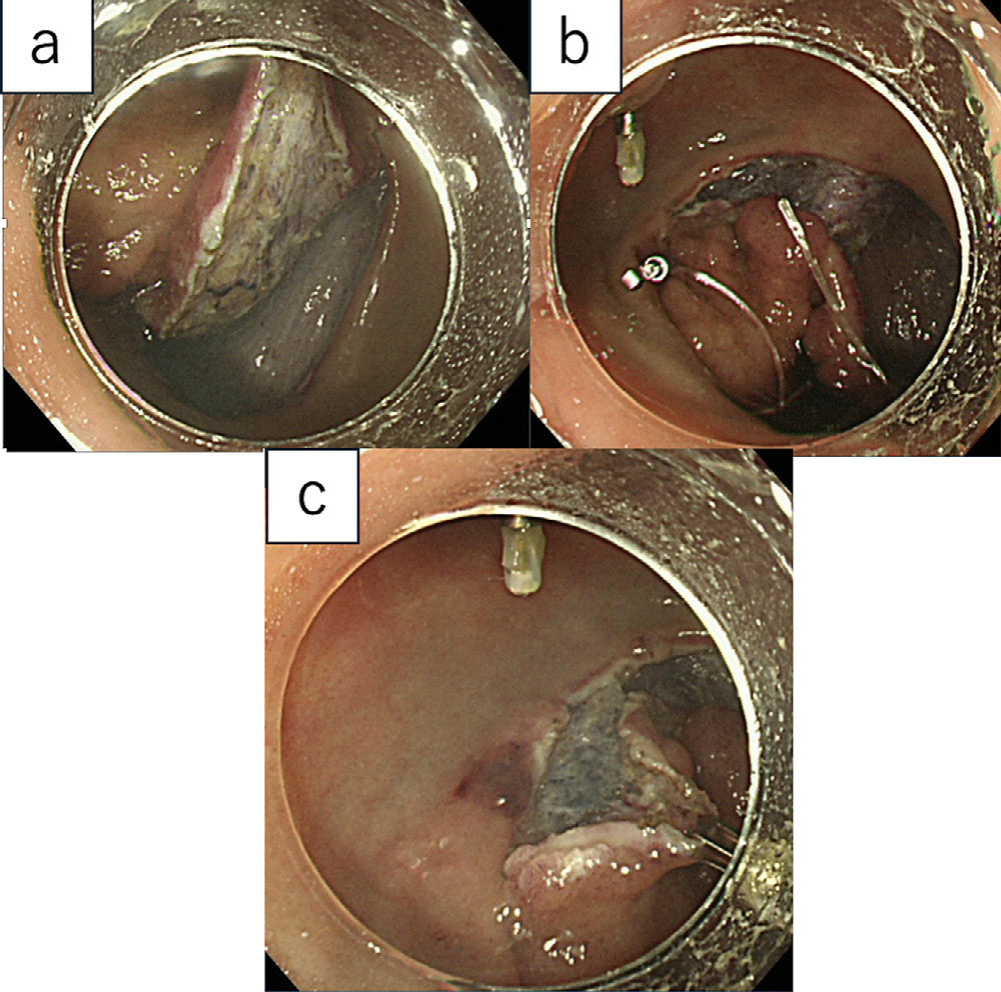
Adjustment of traction direction in the rubber‐clip method. (a–c) The rubber band is cut, and the clip is reattached to a different site to change the direction of traction.

Compared with previous reports using rubber bands for traction, the present study used a polyurethane rubber band, which can be cut easily with alligator forceps. By cutting the band and reapplying the rubber clip, changing traction direction or strengthening traction is simpler and more flexible than with previously reported methods [18, 19] (Figure 3). Operon rubber bands, which are made of polyurethane‐based material, are easier to cut than conventional latex orthodontic rubber bands. Given its low cost, simplicity, and capability for flexible adjustment of traction direction, the RCM appears to be a valuable traction technique for routine use in colorectal ESD.

Regarding rubber band specifications, the 0.3‐mm, 100% spandex Operon thread provides sufficient elasticity for effective traction while remaining thin enough to loop around a standard hemoclip without impeding deployment through the working channel. At 300 JPY per 20‐m roll, the per‐procedure material cost is negligible. Appropriate sizing is important: overly thick bands may hinder clip attachment or delivery, whereas overly thin bands may lack adequate elasticity. The 0.3‐mm spandex Operon thread provided a practical balance of traction force and ease of handling in this study.

In addition to its strengths, this study had several practical challenges. Variability in endoscopist experience may have influenced the application of traction and procedural outcomes. In addition, consistent assessment of factors such as maneuverability and fibrosis was challenging because these evaluations are partly subjective. To address these issues, standardized definitions and predefined evaluation criteria were applied as much as possible.

This study has several limitations. First, this was a single‐center retrospective analysis; therefore, selection bias and unmeasured confounding factors cannot be completely ruled out. Differences in endoscopist skill level may have also influenced the outcomes. Second, the sample size of the propensity score‐matched cohort (56 RCM vs. 56 non‐RCM cases) was relatively small, which may limit the statistical power and generalizability of the findings. Third, use of the RCM may have been concentrated among specific endoscopists, introducing the possibility of endoscopist‐related bias. Fourth, the assessment of endoscopic maneuverability was inherently subjective, and the possibility of inter‐operator variability could not be fully eliminated. Fifth, the rubber band used is not originally approved as a medical device; therefore, our findings do not guarantee its universal safety or efficacy. Sixth, because the RCM was introduced in mid‐2016, the non‐RCM group predominantly consists of earlier cases; institutional and individual learning curves naturally favor the RCM group, and this chronological confounding cannot be entirely eliminated despite propensity score matching. These limitations should be considered when interpreting the results, and further prospective multicenter studies are warranted.

In conclusion, the RCM has potential as a novel traction method in colorectal ESD, contributing to shorter procedure times and safer submucosal dissection.

## Author Contributions

Drafting of the manuscript: **Kohei Uyama**; critical revision of the manuscript: **Hiroyoshi Iwagami**, **Riki Sakano**, **Tomoya Kitada**, **Masayuki Shimoyama**, **Tomoko Terashita**, **Yasuki Nakatani**, **Yoshito Uenoyama**, and **Yukitaka Yamashita**. All authors have read and approved the final version of the manuscript.

## Funding

The authors have nothing to report.

## Ethics Statement

This study protocol was approved by the Institutional Review Board (Approval No. **1608**).

## Consent

Given the retrospective study design, the requirement for individual informed consent was waived by the institutional review board, and an opt‐out method was employed.

## Conflicts of Interest

The authors declare conflicts of interest.
